# Bayesian optimization for seed germination

**DOI:** 10.1186/s13007-019-0422-z

**Published:** 2019-04-29

**Authors:** Artyom Nikitin, Ilia Fastovets, Dmitrii Shadrin, Mariia Pukalchik, Ivan Oseledets

**Affiliations:** 1 CDISE, Skolkovo Institute of Science and Technology, Nobelya 3, Moscow, Russia 121205; 2 V.V. Dokuchaev Soil Science Institute, Pyzhyovskiy lane 7 bld. 2, Moscow, Russia 119017

**Keywords:** Seed germination, Machine learning, Gaussian process, Bayesian optimization, Agriculture

## Abstract

**Background:**

Efficient seed germination is a crucial task at the beginning of crop cultivation. Although boundaries of environmental parameters that should be maintained are well studied, fine-tuning can significantly improve the efficiency, which is infeasible to be done manually due to the high dimensionality of the parameter space.

**Results:**

Traditionally seed germination is performed in climatic chambers with controlled environmental conditions. In this study, we perform a set of multiple-day seed germination experiments in the controllable environment. We use up to three climatic chambers to adjust humidity, temperature, water supply and apply machine learning algorithm called Bayesian optimization (BO) to find the parameters that improve seed germination. Experimental results show that our approach allows to increase the germination efficiency for different types of seeds compared to the initial expert knowledge-based guess.

**Conclusion:**

Our experiments demonstrated that BO could help to identify the values of the controllable parameters that increase seed germination efficiency. The proposed methodology is model-free, and we argue that it may be useful for a variety of optimization problems in precision agriculture. Further experimental studies are required to investigate the effectiveness of our approach for different seed cultures and controlled parameters.

**Electronic supplementary material:**

The online version of this article (10.1186/s13007-019-0422-z) contains supplementary material, which is available to authorized users.

## Introduction

Seed germination has been an interesting subject of study for many years. On the one hand, it is the topic for basic research since many biochemical processes occur during dormancy and different stages of seed germination. On the other hand, the problem is also of great practical importance: finding the optimal parameters such as substrate material, amount of water supply, air temperature, the proportion of plant growth promoters, etc. is a challenging task. Seed germination comprises many processes, and relationships of factors affecting termination of seed dormancy are very diverse. For example, the aforementioned water and temperature combined with light and nitrate level influence seed germination, however, their effect does depend on the level of dormancy of the seeds [1].

The problem becomes even more challenging when multiple parameters must be considered together, and specific sets of parameters are supposed to be optimized for each time step. Dynamic models of seed germination have been developed [1–3] to address this issue. These models may be helpful in understanding the underlying processes of seed germination. However, to achieve satisfactory optimization results using model-based techniques, comprehensive prior knowledge of the problem structure is required [4]. Moreover, particular dynamic models may not be appropriate for the specific conditions that these models were not developed for, e.g., different plant species, substrates or growth stimulators.

A more adaptive approach, based on machine learning (ML) methods, seems to be promising to tackle this issue. Among those methods the *Bayesian optimization* (BO) [5, 6] algorithm based on the *Gaussian process regression* (GPR) is one of the most attractive. It is a black-box optimization algorithm that does not require knowledge of the system intrinsics. It is widely used in the ML community for hyperparameter optimization and was even successfully applied in culinary arts [7]. Similarly, an approach based on Genetic Algorithms and GPR has been previously proposed for precision agriculture [8].

In this paper, we apply BO to simplified seed germination process in the controllable environment in order to identify the values of the controlled parameters that yield the best germination efficiency. First, we select the number of tunable parameters that we can control during the germination period (several days) with the help of climatic chambers, e.g., humidity, temperature, amount of water supply provided and choose the reasonable bounds for these parameters based on the expert knowledge. Then, we iteratively apply BO algorithm, to find the values of parameters that maximize the number of germinated seeds. We show that starting with an initial expert knowledge-based guess our approach allows to find such values of parameters that yield solid improvement both when initial germination efficiency is low (first experiment) and high (second experiment).

## Materials and methods

**Fig. 1:**
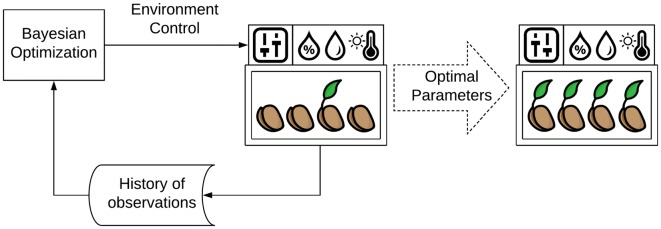
System overview

In this section, we describe the methodology and the algorithms used to build our framework. Figure 1 shows a schematic overview of the proposed system.

### Seed germination

We conducted two experiments, first, using pea seeds (*Pisum sativum* L.) and, second, using radish seeds (*Raphanus sativa* L.) in different settings. Seeds were purchased from Federal Scientific Center of Vegetable (Odintsovo, Russia). The weight of 100 seeds showed an average of $0.751 \pm 0.01 \, {\hbox {g}}$ for radish, and of $19.95 \pm 1.31 \, {\hbox {g}}$ for pea. All seeds were presterilized in 0.5% of KMnO4 solution for 10 min and then rinsed for several times with deionized water. Three climatic chambers (Binder KBWF 240, KBF 240, KMF 240) allowed to control air temperature ($\pm 0.1 \, ^{\circ }{\hbox {C}}$) and humidity ($\pm \, 1\%$), which was maintained at 80%. No light sources were used in the chambers during the experiments.

The first experiment was conducted in the form of sequential trials with each trial comprising three concurrent germination processes and lasting for 72 h (3 days in total). One hundred pea seeds were placed on a dish covered with sterile cheesecloth and put in each of the three climate chambers to germinate. Totally, 7 controllable parameters were selected: air temperature and the amount of water supplied at 0, 24, 48, 72 and 0, 24, 48 h steps, respectively. The temperature in the chambers was changed smoothly between the selected values during the trials.

**Fig. 2:**
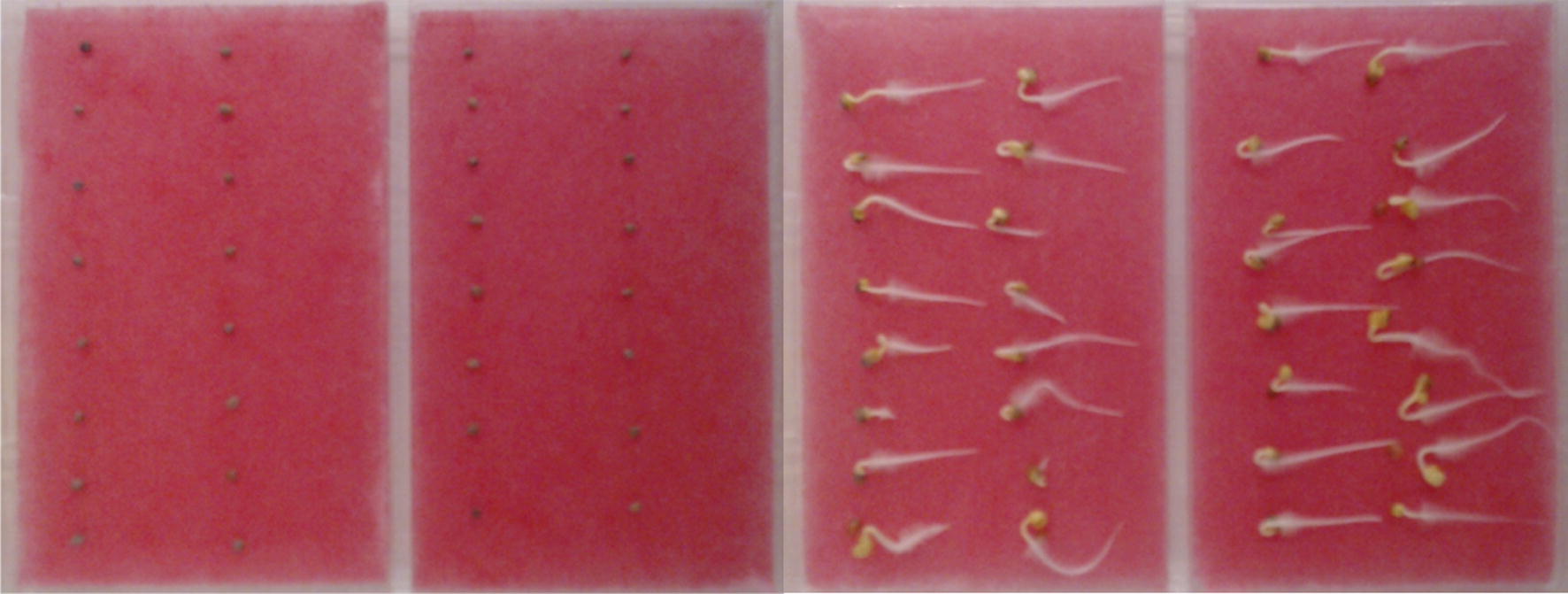
Container with radish seeds before germination (left) and after (right)

During the second experiment, only two climatic chambers were used (KBF 240, KMF 240) to set 4 controllable parameters, namely temperatures at 0, 12, 24, 36 h. Seeds were placed in containers of size $21 \times 15.5 \times 0.8 \, {\hbox {cm}}$ with two sections (each accommodating 16 seeds) on the cloth and watered once at the beginning of a trial with a fixed amount of 6 ml. Figure 2 depicts a single container at the beginning (left) and the end (right) of a trial.

**Fig. 3:**
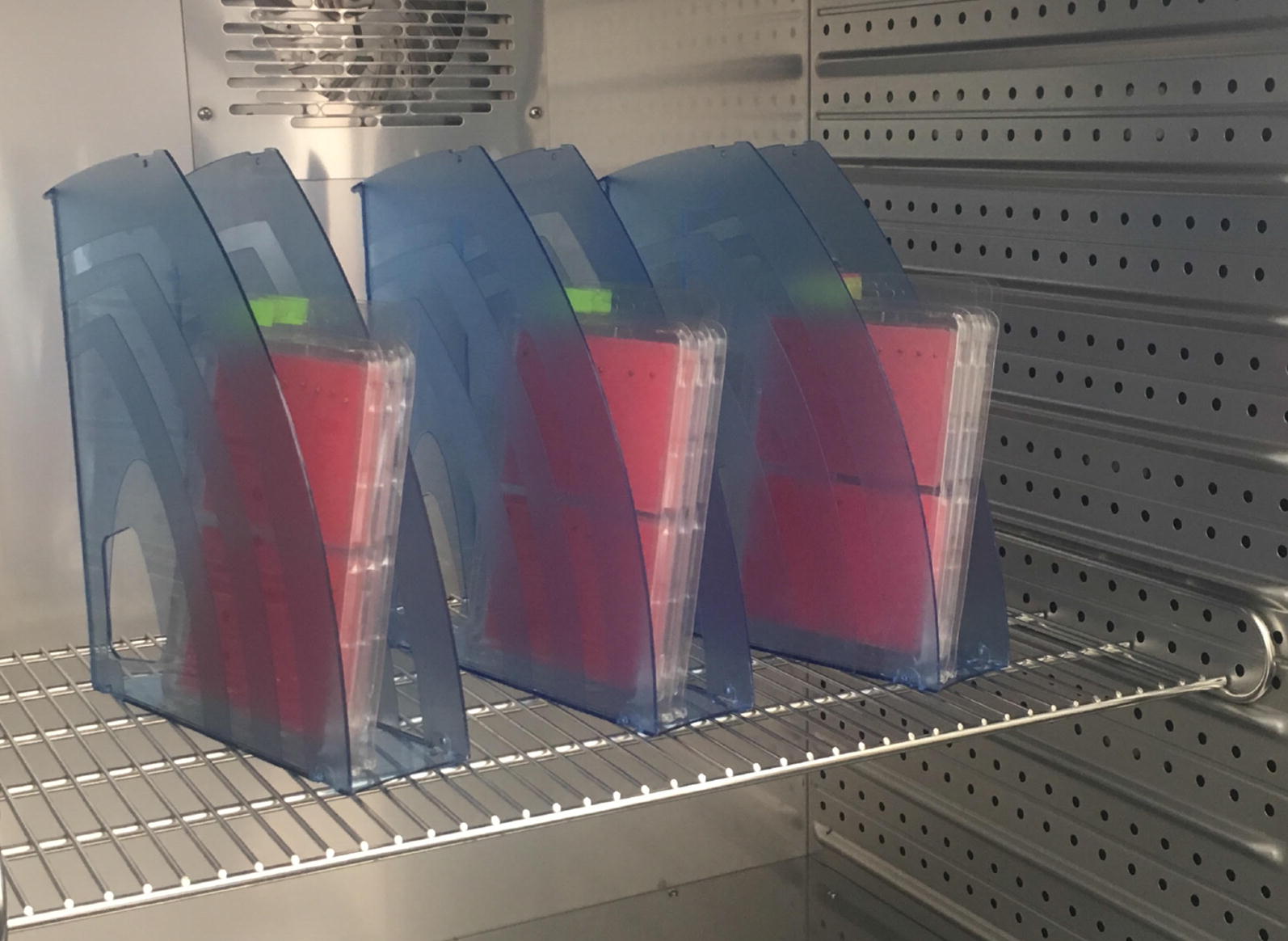
Chamber with 3 groups of 3 containers installed during a single trial in the second experiment

These containers, then, were grouped by 3, giving 96 seeds in a group. Three such groups then were placed almost vertically in each of two climatic chambers with the same controllable parameters set, thus, for each trial giving 6 repetitions with a total amount of seeds equal to 96 in each of them. Figure 3 shows how containers with seeds were installed in the chambers during the second experiment.

**Fig. 4:**
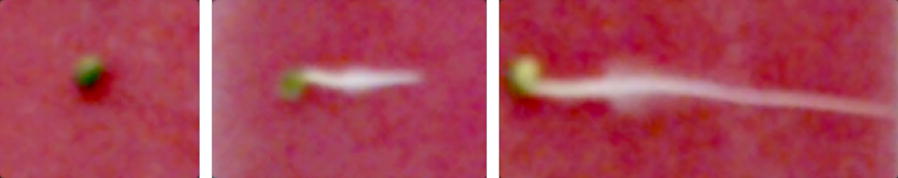
Example of not germinated (left), germinated (middle, 12 mm) and well-germinated (right, 38 mm) radish seeds in the second experiment at the same scale

After the seeds were germinated, the number of germinated and well-germinated seeds were counted in each chamber. In the first experiment, we considered the seeds germinated when only the radicle emerged and could be visibly separated from the seed. If not only radicle but also the hypocotyl emerged and could be visibly separated, the seed was classified as well-germinated. For the second experiment, we considered seeds germinated if radicle emerged and its length is less than 17.5 mm, and well-germinated if it is larger. Figure 4 shows an example of not germinated (left), germinated (middle) and well-germinated (right) radish seeds according to our methodology.

### Bayesian optimization framework

In this section, we describe the Bayesian optimization framework based on the Gaussian process regression that we used in our work.

#### Gaussian process regression

Bayesian optimization relies on the Gaussian Process Regression [9], also called *kriging* in geostatistics, which learns a generative probabilistic model of an arbitrary function of independent variables with the assumption of normality. A Gaussian process is completely determined by its *mean* $\mu (\cdot )$ and *covariance* (kernel) $k(\cdot , \cdot )$ functions:

$$\begin{aligned} f({\mathbf {x}}) & \sim {\mathcal {GP}}\left( m({\mathbf {x}}), k\left( {\mathbf {x}}, {\mathbf {x}}^\prime \right) \right) , \\ m({\mathbf {x}}) &= {\mathbb {E}}~f({\mathbf {x}}), \\ k({\mathbf {x}},{\mathbf {x}}^\prime ) & = {\mathbb {E}}~\left[ \left( f\left( {\mathbf {x}}\right) - m\left( {\mathbf {x}}\right) \right) \left( f\left( {\mathbf {x}}^\prime \right) - m\left( {\mathbf {x}}^\prime \right) \right) \right] , \end{aligned}$$

where ${\mathbf {x}}\in {\mathbb {R}}^d$ is a vector of *d* input parameters.

Let consider the GP model with an additive normal noise:

1 $$\begin{aligned} y({\mathbf {x}}) = f({\mathbf {x}}) + \epsilon , \end{aligned}$$

where $\epsilon \sim {\mathcal {N}}(0, \sigma ^2)$. Given the training data ${\mathbf {X}}=\left( {\mathbf {x}}_1,\ldots ,{\mathbf {x}}_n\right) ^\intercal \in {\mathbb {R}}^{n \times d}$, ${\mathbf {y}}=\left( y_1,\ldots ,y_n\right) ^\intercal \in {\mathbb {R}}^n$, where *n* is the number of available measurements and $(\cdot )^\intercal$ denotes the transpose, the predictive distribution at an unobserved point ${\mathbf {x}}^*$ is given by

$$\begin{aligned} f^* & \sim {\mathcal {N}}\left( {\hat{\mu }}, {\hat{\sigma }}^2\right) , \\ {\hat{\mu }}({\mathbf {x}}^*) & = m({\mathbf {x}}^*) + K({\mathbf {x}}^*, {\mathbf {X}})[K({\mathbf {X}},{\mathbf {X}}) + \sigma ^2 I] ({\mathbf {y}}- m({\mathbf {X}})), \\ {\hat{\sigma }}^2({\mathbf {x}}^*) &= k({\mathbf {x}}^*, {\mathbf {x}}^*) - K({\mathbf {x}}^*, {\mathbf {X}})[K({\mathbf {X}},{\mathbf {X}}) + \sigma ^2I]^{-1}K({\mathbf {X}}, {\mathbf {x}}^*), \end{aligned}$$

where $K({\mathbf {X}}, {\mathbf {X}})$ is a matrix of the form $K_{ij} = k({\mathbf {x}}_i, {\mathbf {x}}_j), i,j=1,\ldots ,n$. Particular choice of the kernel function depends on the assumptions about the model and a particular application, however, there exist commonly used kernels, such as *Radial basis function* (RBF) and Mateŕn that work well in general. Kernel hyperparameters are usually optimized using *Maximum Likelihood Estimation* (MLE) [10] or its variations.

**Fig. 5:**
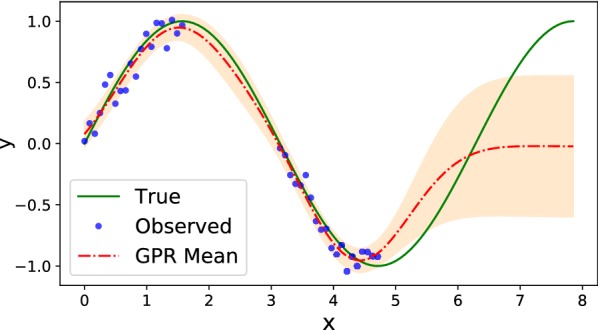
Gaussian process regression (red dashed line depicts the predictive mean and orange fill depicts the standard deviation intervals) with noisy measurements (blue dots) of the sine function (solid green line) using RBF kernel. The predictive variance increases in the areas of missing measurements, and the predictive mean fails to capture the true function trend outside of the interpolation region

Figure 5 shows an example of GPR using RBF kernel over the sine function with noisy measurements, where predictive variance increases at points with missing measurements. Outside of the interpolation region predictive variance significantly increases with the mean failing to capture the true function trend.

#### Bayesian optimization

An advantageous property of GPR is that it provides not only the prediction of the value at unobserved points but the complete probabilistic distribution determined by the mean and variance. The general idea behind BO algorithms is to use such distribution to explore parameter space and select values of ${\mathbf {x}}^*$ in a way that it will most probably maximize target function $f({\mathbf {x}})$. The common approach is to select a particular *acquisition function* that takes parameters of the predictive distribution of the fitted model as an input and outputs some value which is maximized instead. There exist multiple strategies, for example, using the *probability of improvement*, *expected improvement* or *integrated expected improvement* over the current best value, *entropy search* or *upper confidence bound* (UCB) [6]. We have selected the UCB acquisition function in our work as it is easy to evaluate and was shown to be effective in practice. It is expressed using the predictive mean and variance as follows:

2 $$\begin{aligned} a_{UCB}({\mathbf {x}}, \kappa ) = {\hat{\mu }}({\mathbf {x}}) + \kappa \cdot {\hat{\sigma }}({\mathbf {x}}) \end{aligned}$$

Exploration–exploitation trade-off is managed by the parameter $\kappa$, where for small $\kappa$ regions with a high mean (exploitation) and large $\kappa$ regions with high uncertainty (exploration) are preferred, respectively. We will further omit $\kappa$ from the arguments of the UCB function where it is assumed fixed.

**Fig. 6:**
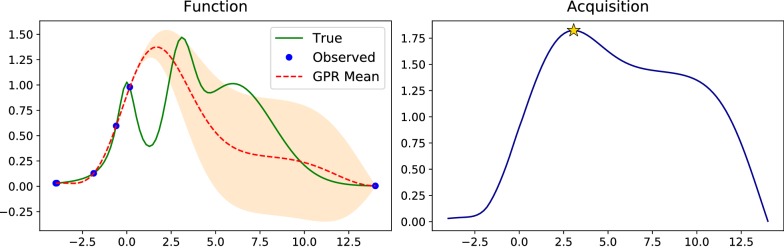
The fourth step of the Bayesian optimization procedure with $\kappa =2$. Left: optimized function (solid green line), observed points (blue dots), GPR predictive mean (dashed red line) and standard deviation intervals (orange fill). Right: UCB acquisition function at the current step with star depicting the next guess, which is close to the true maximum

Figure 6 shows the 4th step (with 2 initial data points at the boundaries) of the BO algorithm on an example function with several local maximums using UCB acquisition function with the fixed $\kappa =2$.

It is critical to note that BO performance is profoundly affected by the dimensionality of the input data due to the exponential growth of the parameter space. It may start to perform poorly when the number of controlled parameters becomes larger than ten [11].

### Noise estimation

We defined the target function that we aim to optimize as the sum of averages of germinated and well-germinated seeds (see “Seed germination” section). First, let *N* denote the number of seeds used in the experiment. Second, due, to the stochasticity, we model the success of a single seed germination for the fixed values of parameters ${\mathbf {x}}$ as a Bernoulli trial. Then, the probability that a single seed is germinated equals to $p({\mathbf {x}})=p$, whereas probability that a single seed is well-germinated, given that it has germinated, equals to $q({\mathbf {x}}) = q$. If $N_g$ and $N_{wg}$ denote the number of germinated and well-germinated seeds in the experiment, respectively, then, it can be shown that for sufficiently large *N* (for details, see “Appendix” section) our target function is

$$\begin{aligned} y({\mathbf {x}}) = \frac{N_g + N_{wg}}{N}~\sim ~{\mathcal {N}}\left( \mu , \frac{1}{N}\sigma ^2\right) , \end{aligned}$$

where $\mu =p(1+q)$ and $\sigma ^2=p(1+3q) - p^2(1+q)^2$. Due to the normality of the obtained distribution, its variance can be interpreted as an input-dependent Gaussian noise in the Eq. (1). Therefore, we can simplify hyperparameter optimization by setting a lower bound of the noise variance with the following value:

3 $$\begin{aligned} \frac{1}{N} \max _{p,q} \sigma ^2(p,q) = \frac{1}{N}. \end{aligned}$$

Alternatively, for each obtained observation $y_i$ a lower-bound of the noise variance can be estimated as (for details, see “Appendix” section)

$$\begin{aligned} \frac{1}{N} \cdot y_i(2-y_i), \quad i=1, \ldots ,n \end{aligned}$$

in order to incorporate the dependence on the values of observations.

### Concurrent experiments

Aforementioned BO formulation assumes that the optimization process is sequential, i.e., only a single $x^*$ is selected at each step. However, it may be necessary to be able to select several vectors of parameters to explore, e.g., if there are multiple CPU cores for computations or several experimental setups available (climate chambers in our case). This is referred in the literature as batch setting [12, 13] or setting with a delayed feedback [14]. In this work we consider the following approach from [12] to tackle this problem: for each trial comprising the selection of multiple vectors of parameters, we find the maximizer of acquisition function and “observe” the target function using the predictive mean of GPR instead of the real outcome (see Algorithm 1). 
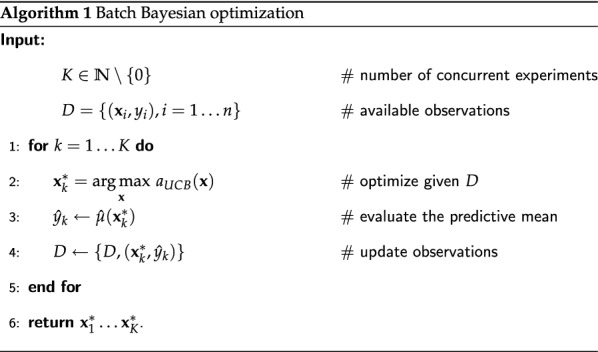


### Exploration–exploitation control

It may happen when performing exploitation that the algorithm could propose parameters that are very close to the already explored data points, e.g., try $22.001 \, ^{\circ }{\hbox {C}}$ temperature after $22.000 \, ^{\circ }{\hbox {C}}$, which yields a change beyond the controllable precision. In order to cope with this problem and reduce the manual labor of an operator in the selection of $\kappa$ from Eq. (2) that will give a reasonable exploitation, we propose an additional optimization procedure. First, we formulate the notion of a reasonable exploitation as the following constraint:

4 $$\begin{aligned} \underset{i=1, \ldots ,n}{\min } \left\| \underset{{\mathbf {x}}}{\arg \max }~a_{UCB}({\mathbf {x}}, \kappa ) - {\mathbf {x}}_i\right\| _\infty \ge \epsilon _{{\textit{xploit}}}, \end{aligned}$$

where *n* is the number of already observed data points and $\epsilon _{{\textit{xploit}}}$ is a predefined constant. This constraint means that at least one of the parameters must be at least as far as $\epsilon _{{\textit{xploit}}}$ from the respective parameter of the closest already observed data point. One can think of a more fair constraint, where a too small change of a parameter is diminished to zero, however, it may pose challenges for the optimization algorithms. Similarly, in order to avoid unreasonable exploration, we consider the following constraint:

5 $$\begin{aligned} \underset{i=n_1, \ldots ,n_s}{\min } \left\| \underset{{\mathbf {x}}}{\arg \max }~a_{UCB}({\mathbf {x}}, \kappa ) - {\mathbf {x}}_i\right\| _1 \le \epsilon _{{\textit{xplore}}}, \end{aligned}$$

where ${\mathbf {x}}_i$ is taken from a subset of size $s \le n$ of already observed points, e.g., one may like to ignore manually initialized data (see “Data preparation” section) and prefer exploration around knowingly good regions. This constraint means that the selected parameters must be at most as $\epsilon _{{\textit{xplore}}}$ far in total form the closest already observed data point. Algorithm 2 describes the exploration–exploitation control procedure. 
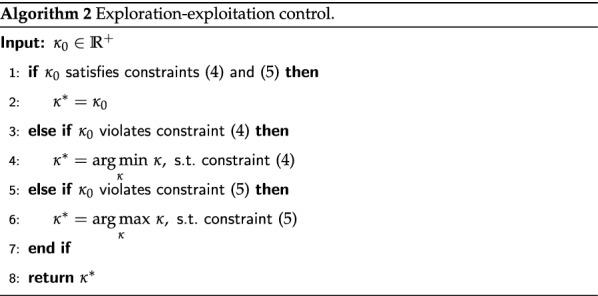


## Experimental evaluation

In this section, we describe the details of our experimental setup and provide the obtained results.

### Selecting parameters

We implemented^1^ our solution with *Python 3* programming language using the Bayesian optimization library.^2^ As the covariance function we selected the composition of constant, isotropic Mateŕn (with $\nu =2.5$, assuming sufficient smoothness) and white noise kernels with tunable hyperparameters:

$$\begin{aligned} k({\mathbf {x}}_i,{\mathbf {x}}_j) = \alpha \cdot C_\nu ({\mathbf {x}}_i / \rho , {\mathbf {x}}_j / \rho ) + \sigma ^2 \delta _{ij} \end{aligned}$$

where $\delta _{ij}$ is a Kronecker-delta, $\alpha , \rho \in {\mathbb {R}}^{+}$. Optimization of the hyperparameters is performed at each step when new data is being available using the MLE with the number of optimizer restarts equal to 30. Bounds for hyperparameter optimization were set as follows: $\alpha \in [10^{-5},10^5]$, $\rho \in [10^{-5}, 10^5]$ and $\sigma ^2 \in [0.01, 10^5,]$ (see “Seed germination” and “Noise estimation” sections). GP mean was selected to be the mean value of the observed measurements.

Given the small number of tunable parameters (7 in the first experiment and 4 in the second), we considered the basic BO approach. As an acquisition function, we selected UCB since it has been shown to be effective in various scenarios. Exploration–exploitation trade-off was managed through $\kappa$ parameter based on the expert knowledge, i.e., at each step, $\kappa$ was selected in such a way that the algorithm does not purely exploit almost the same parameters or explore knowingly unprofitable regions. Additional control was performed by setting $\epsilon _{{\textit{xploit}}}$ equal to $0.1 \, ^{\circ }{\hbox {C}}$ and 1 ml and $\epsilon _{{\textit{xplore}}}$ equal to $10 \, ^{\circ }{\hbox {C}}$ and 100 ml for the temperature and the water supply, respectively. For constrained optimization we have used *SciPy* [15] library implementation of the *Sequential least squares programming* (SLSQP) algorithm [16]. Each optimization step requires the evaluation of the maximum of acquisition function at several points, which impose computational overhead, however, it can be considered negligible compared to the time-scale of a single trial.

### Data preparation

To set up the experiments, we had to consider several issues. First, we had to select the boundaries for the optimized parameters: we selected them at 0, $40 \, ^{\circ }{\hbox {C}}$ (in both experiments) and 0, 250 ml (in the first experiment) for the temperature and the water supply, respectively. Second, as the parameters may have different unit measures, which affects modeling due to isotropy of the selected kernel, we needed to scale them appropriately: we linearly mapped temperature and water supply values to [0, 1] and [0, 0.5] intervals, respectively, assuming “equivalence” of $1 \, ^{\circ }{\hbox {C}}$ and 12.5 ml (during the second experiment, this step was ignored as the only temperature was varied). Finally, we had to add some initial data so that optimization could kick off: we picked all of the possible combinations of 0 and 40 temperatures (in both experiments) with 0 water supply (in the first experiment) on each day and assigned the “observed” target function values equal to 0 (totally $2^4=16$ initial points). It can be considered reasonable as extreme conditions should produce poor results.

### Results

#### First experiment (poorly germinated pea seeds)

For a single germination process, we used $N=100$ pea seeds and conducted only a single repetition for each selected vector of controlled parameters. The first trial was conducted using the single reference vector of parameters selected with the expert knowledge, which gave the number of germinated seeds equal to 73, and the two vectors selected by the BO algorithm. At the 11th observation the algorithm discovered the parameters, which yielded 73 germinated seeds with an additional amount of 18 well-germinated. The 20th selected vector of parameters produced as much as 80 germinated and 33 well-germinated seeds, which in total gave a 55% improvement over the initial guess. Subsequent 13 steps didn’t provide any further enhancement.

**Fig. 7:**
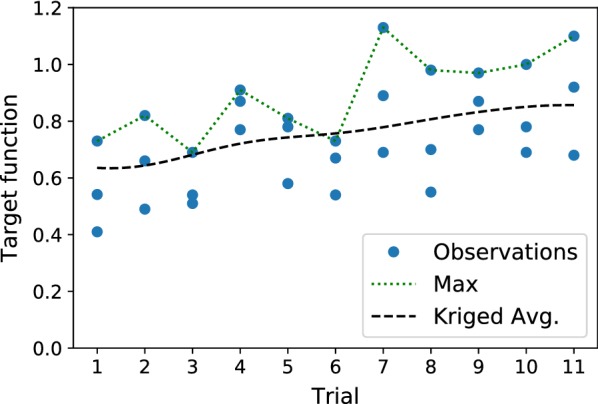
Target function values (blue dots) for each chamber during 11 trials, the maximum in each trial (green top dotted line) and the kriged mean (black dashed line). The highest germination efficiency is achieved at the 7th trial with 80 germinated and 33 well-germinated seeds

Figure 7 shows the target values obtained during 11 trials of the first experiment. Black dashed line denotes the kriged average and shows the trend of improvement in the germination efficiency, whereas the green top dotted line shows the best-observed values for each trial. Table 1 depicts all of the 33 vectors of parameters and respective observed target function values obtained during 11 trials.

**Table 1:** Values of the 33 explored vectors of parameters $\left( {\mathbf {t}}_1,\ldots , {\mathbf {t}}_4, {\mathbf {w}}_1,\ldots , {\mathbf {w}}_3\right) ^T$ and respective target function values

#	${\hbox {t}}_1$	${\hbox {t}}_2$	${\hbox {t}}_3$	${\hbox {t}}_4$	${\hbox {w}}_1$	${\hbox {w}}_2$	${\hbox {w}}_3$	Target
1	24.7	21.5	24.2	22.6	200	200	200	0.73
2	24.6	19.8	24.5	24.1	200	195	213	0.41
3	25.9	22.0	25.1	22.0	200	182	203	0.69
4	24.9	21.4	24.2	23.0	200	200	197	0.49
5	25.1	24.8	26.8	27.8	196	3	179	0.82
6	23.0	21.3	27.8	26.6	187	208	206	0.66
7	24.7	21.6	24.3	22.4	200	197	207	0.69
8	25.0	22.5	24.3	21.4	200	195	202	0.51
9	20.6	26.7	29.3	28.8	178	9	179	0.54
10	25.1	24.8	26.8	27.8	196	3	179	0.87
11	21.7	26.2	28.6	28.6	182	8	179	0.91
12	26.5	27.7	25.7	26.6	159	10	169	0.77
13	21.6	26.3	28.7	28.6	182	8	179	0.81
14	25.6	21.7	24.3	22.1	204	189	201	0.58
15	26.0	21.8	25.8	26.8	195	178	227	0.78
16	28.2	24.1	26.6	28.9	228	18	235	0.73
17	27.5	24.7	24.0	30.1	239	5	248	0.54
18	30.4	21.0	34.3	23.7	241	0	250	0.67
19	26.4	22.2	29.9	25.6	170	39	144	0.89
20	*25*.*8*	*23*.*7*	*29*.*6*	*25*.*2*	*173*	*39*	*162*	*1*.*13*
21	25.8	25.7	30.2	25.5	249	45	108	0.69
22	22.4	22.4	32.4	25.2	127	52	201	0.7
23	23.3	22.3	31.6	26.0	139	52	188	0.98
24	21.0	20.6	30.1	23.2	146	30	186	0.55
25	24.0	23.9	32.7	24.5	125	61	194	0.87
26	24.2	23.2	31.8	25.0	136	54	184	0.97
27	25.7	24.7	32.7	26.6	138	73	188	0.77
28	22.4	20.8	29.6	23.3	147	18	164	0.78
29	23.0	21.6	29.8	24.0	150	27	166	1.0
30	22.7	22.5	29.8	22.7	156	26	172	0.69
31	24.0	21.6	28.9	23.6	160	28	157	0.68
32	24.1	22.0	29.0	23.9	162	32	160	1.1
33	24.0	22.4	29.0	23.1	167	32	165	0.92

Parameters ${\mathbf {t}}$ and ${\mathbf {w}}$ stand for the air temperature in $^{\circ }{\hbox {C}}$ and the water supply in ml, respectively. The optimal parameters are highlighted in italics

Notably, without any prior knowledge of the underlying system, the algorithm was able to learn the values of the controlled parameters that yield sufficient improvement of the germination efficiency. The values of the parameters that achieved the maximum found target function value of 1.13 at the 20th iteration are listed in italics in Table 1. The identified values can be explained from the physiological point of view. For example, periodically changing temperature may be favorable due to the natural adaptation of seeds to day and night, whereas water supply identified by the algorithm is in a good agreement with the dynamics of water uptake by seeds, previously described in [17]. According to this study, water uptake by plant seeds is triphasic, comprising a rapid initial absorption, followed by a plateau phase and a further increase due to embryonic axes elongation.

#### Second experiment (well-germinated radish seeds)

Although the first experiment showed a substantial improvement of germination efficiency in the case of poorly germinated seeds, it could not be that easily observed for well-germinated seeds. Therefore, in the second experiment, we used $N=96$ radish seeds with 6 repetitions for a single germination trial. The first 4 trials were conducted by setting all of the temperature parameters as either 21, 22, 23 or 24. At the 9th trial (5th automatic step), the algorithm discovered the parameters, which yielded the best average of 10 germinated and 88 well-germinated seeds.

**Fig. 8:**
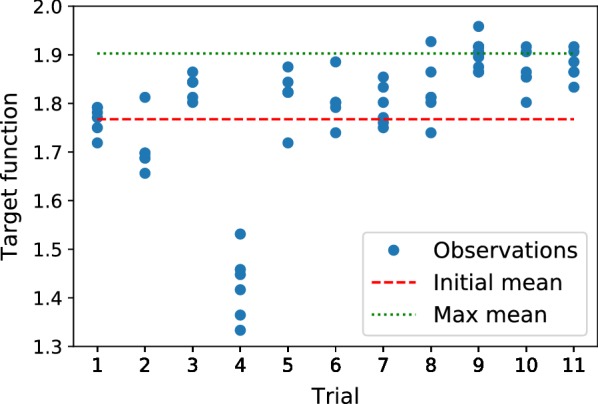
Target function values (blue dots) for each vector of parameters, mean of the initial expert-knowledge guess (red dashed line) and the best found mean for the 9th vector (green dotted line) with around 10 germinated and 88 well-germinated seeds

Figure 8 shows the target values obtained during 12 trials, where the last trial served as a validation for the best found vector of parameters during the 9th trial. Green dotted line shows the best-observed mean value of the target function, whereas the red dashed line depicts the first expert-knowledge guess-based trial.

Table 2 lists all of the 11 vectors of parameters and the corresponding means and standard deviations of the target function values obtained during 12 trials. The complete table containing target function values for every repetition during each trial can be found in Additional file 1.

**Table 2:** Values of the 11 explored vector of parameters $\left( {\mathbf {t}}_1,\ldots , {\mathbf {t}}_4\right) ^T$ and the corresponding mean and standard deviation values of the target function

#	${\hbox {t}}_1$	${\hbox {t}}_2$	${\hbox {t}}_3$	${\hbox {t}}_4$	Mean	SD
1	23.0	23.0	23.0	23.0	1.767	0.028
2	22.0	22.0	22.0	22.0	1.707	0.054
3	24.0	24.0	24.0	24.0	1.835	0.023
4	21.0	21.0	21.0	21.0	1.425	0.071
5	23.9	23.9	27.1	23.9	1.818	0.053
6	21.7	27.7	25.2	21.7	1.804	0.047
7	19.7	25.0	25.1	26.5	1.795	0.042
8	21.7	24.8	25.3	23.6	1.826	0.063
9	*23*.*0*	*27*.*7*	*22*.*3*	*25*.*6*	*1*.*903*	*0*.*026*
10	23.3	26.9	23.0	25.3	1.866	0.041
11	20.7	33.2	20.7	25.8	1.878	0.031

Parameters ${\mathbf {t}}$ stand for the air temperature in $^{\circ }{\hbox {C}}$. The optimal parameters are highlighted in italics

Although with the initial guess seeds already propagated efficiently, the algorithm was able to achieve substantial improvement after the several steps and identify the parameters, which yielded the maximum mean value of 1.903 of the target function with low dispersion.

## Conclusions and future work

We applied Bayesian optimization framework to the seed germination process in a controlled environment. Our experiments demonstrated that the proposed methodology allowed to identify the values of the controllable parameters that increase germination efficiency in different settings for different seeds both in the case when initial expert-knowledge based guess yields low and high germination efficiency. The proposed methodology is model-free, and we argue that it may be useful for a variety of optimization problems in intelligent agriculture. Using this approach, we achieved increase in germination efficiency (according to our metrics) from 36.5 to 56.5% by 19 iterations in the first experiment (pea seeds) with low initial germination efficiency, whereas in the second experiment (radish seeds) with high initial germination efficiency the increase was from 91.8% up to 95.2% by 5 iterations.

We note that selection of the controllable parameters must be made carefully during the preliminary planning. On the one hand, increasing their number allows to perform better fine-tuning, on the other hand, it makes BO algorithms less efficient and requires more trials to be conducted, which may be both overly time-consuming and equipment demanding.

Combination of the proposed technique with the existing methods of computer vision-based seed counting [18, 19] and seed quality evaluation [20] may decrease manual labor significantly and improve scalability. The BO methods definitely could help to reveal optimum chemical parameters of growing mediums or find the environmentally friendly doses of plants biostimulants (humic substances, synthetic hormones, etc.), which effects on plants usually have a nonlinear dose-effect relationship. Further experimental studies are required to investigate the effectiveness of our approach for this environmental and plants issues. Additionally, we aim to consider partially-controllable environments and apply the proposed method at the next stages of plant growth.

### Additional file

**Additional file 1.** Radish seeds experiment data. The complete list of 11 explored vectors of parameters and target function values obtained during 12 trials of the second experiment with radish seeds.

## Appendix

Let random variable $x \sim B(1, p)$ denote the success of a seed germination with a probability *p* and $y | x=1 \sim B(1, q)$ denote the success of a well-germination with a probability *q* given that germination occurred. Using the formula for a full probability:

$$\begin{aligned} p_{x,y}(x=0, y=0) &= 1-p \\ p_{x,y}(x=1, y=0) &= p(1-q)\\ p_{x,y}(x=0, y=1)&= 0 \\ p_{x,y}(x=1,y=1) &= pq \end{aligned}$$

Then, the distribution of a random variable $z = x + y$ is

$$\begin{aligned} p_z(z=0)&= 1-p\\ p_z(z=1)&= p(1-q)\\ p_z(z=2)&= pq \end{aligned}$$

with the mean and the variance

6 $$\begin{aligned} \mu&=p(1+q), \end{aligned}$$

7 $$\begin{aligned} \sigma ^2&=p(1+3q) - p^2(1+q)^2. \end{aligned}$$

Let $z_i \sim p_Z,~ i=1, \ldots ,N$ be identically independently distributed random variables. Then, according to the Central Limit Theorem, for sufficiently large *N*, the distribution of their average can be well approximated by a normal distribution:

$$\begin{aligned} w = \frac{1}{N}\sum _{i=1}^N {z_i}~\sim ~{\mathcal {N}}_w(\mu , \sigma ^2 / N). \end{aligned}$$

Given *M* samples $w_i \sim {\mathcal {N}}_w,\, i=1,\ldots ,M$ one can find the sampling mean ${\widetilde{\mu }} = (w_1 + \cdots + w_M)/M$ and estimate the variance, by substituting the Eq. (6) into the Eq. (7), as

8 $$\begin{aligned} {\widetilde{\sigma }}^2 = \frac{1}{N}\left[ \frac{1 + 3q}{1 + q} \cdot {\widetilde{\mu }} - {\widetilde{\mu }}^2 \right] \le \frac{1}{N}\cdot {\widetilde{\mu }}(2 - {\widetilde{\mu }}). \end{aligned}$$

## Abbreviations

GPR: Gaussian process regression
RBF: radial basis function
MLE: maximum likelihood estimation
BO: Bayesian optimization
UCB: upper confidence bound

## Footnotes

1 https://github.com/tzoiker/bo-seed-germination/tree/v0.1.

2 https://github.com/fmfn/BayesianOptimization.
